# A Positive Association between *T. gondii* Seropositivity and Obesity

**DOI:** 10.3389/fpubh.2013.00073

**Published:** 2013-12-25

**Authors:** Gloria M. Reeves, Sara Mazaheri, Soren Snitker, Patricia Langenberg, Ina Giegling, Annette M. Hartmann, Bettina Konte, Marion Friedl, Olaoluwa Okusaga, Maureen W. Groer, Harald Mangge, Daniel Weghuber, David B. Allison, Dan Rujescu, Teodor T. Postolache

**Affiliations:** ^1^Child and Adolescent Mental Health Innovations Center, Department of Psychiatry, University of Maryland, School of Medicine, Baltimore, MD, USA; ^2^Division of Child and Adolescent Psychiatry, Department of Psychiatry, University of Maryland, School of Medicine, Baltimore, MD, USA; ^3^St. Elizabeths Hospital, Psychiatry ResidencyTraining Program, Washington, DC, USA; ^4^Division of Endocrinology, Diabetes, and Nutrition, Department of Medicine, University of Maryland, School of Medicine, Baltimore, MD, USA; ^5^Department of Epidemiology and Public Health, University of Maryland, School of Medicine, Baltimore, MD, USA; ^6^Department of Psychiatry, Martin-Luther-Universität Halle-Wittenberg, Halle, Germany; ^7^Department of Psychiatry and Behavioral Sciences, The University of Texas Health Science Center at Houston, TX, USA; ^8^Colleges of Nursing and Medicine, University of South Florida, Tampa, FL, USA; ^9^Research Unit on Lifestyle and Inflammation-associated Risk Biomarkers, Clinical Institute for Medical and Chemical Laboratory Diagnosis, Medical University of Graz, Graz, Austria; ^10^Department of Pediatrics, Paracelsus Medical School, Salzburg, Austria; ^11^Section on Statistical Genetics, Department of Biostatistics, Nutrition Obesity Research Center, University of Alabama at Birmingham, Birmingham, AL, USA; ^12^Mood and Anxiety Disorder Program, Department of Psychiatry, University of Maryland, School of Medicine, Baltimore, MD, USA

**Keywords:** obesity, body weight, *Toxoplasma gondii*, parasitic infection, inflammation

## Abstract

Obesity is a global public health problem that is linked with morbidity, mortality, and functional limitations and has limited options for sustained interventions. Novel targets for prevention and intervention require further research into the pathogenesis of obesity. Consistently, elevated markers of inflammation have been reported in association with obesity, but their causes and consequences are not well understood. An emerging field of research has investigated the association of infections and environmental pathogens with obesity, potential causes of low grade inflammation that may mediate obesity risk. In this study, we estimate the possible association between *Toxoplasma gondii* (*T. gondii*) infection and obesity in a sample of 999 psychiatrically healthy adults. Individuals with psychiatric conditions, including personality disorders, were excluded because of the association between positive serology to *T. gondii* and various forms of serious mental illness that have a strong association with obesity. In our sample, individuals with positive *T. gondii* serology had twice the odds of being obese compared to seronegative individuals (*p* = 0.01). Further, individuals who were obese had significant higher *T. gondii* IgG titers compared to individuals who were non-obese. Latent *T. gondii* infection is very common worldwide, so potential public health interventions related to this parasite can have a high impact on associated health concerns.

## Introduction

Approximately 57% of the world’s adult population is projected to be obese or overweight by 2030 (1), highlighting the pressing need for research on obesity pathogenesis to identify novel targets for intervention. Public perception is that lifestyle practices have “a lot to do” with causing obesity (2), and the American Heart Association emphasizes accurate tracking of diet and activity for weight management (3). However, lifestyle intervention often results in modest and unsustained weight loss (4). Emerging research on etiology of obesity has investigated the potential role of environmental infections, a concept referred to as “infectobesity” (5, 6), as well as gut microbiota (7) in obesity pathogenesis. If the suspected role of infection in obesity is confirmed, then individuals with obesity of this type might be treated by specifically addressing underlying causes rather than relying on a standard diet and activity intervention (8). Moreover, novel prevention strategies may be developed that may supplement the current, relatively stagnant, diet, and activity recommendations.

The first report of virus-induced obesity in an animal model was reported by Lyons et al. (9) after exposing mice to canine distemper virus. Since that time, several viruses have been shown to cause obesity in animal models (6). Herpes simplex viruses 1 and 2, gut microflora, *Helicobacter pyloris*, *Selenomonas noxia*, and *Chlamydophila pneumonia* have been studied for possible association of infection with obesity, with adenovirus 36 (Ad36) being the most studied in humans (10), although a causal role is unclear (11). Yamada et al. (12) reported an association of Ad36 infection with obesity risk and weight gain, but not with abdominal obesity (i.e., increased waist circumference), suggesting that Ad36 infection is preferentially associated with accumulation of subcutaneous fat as opposed to visceral fat. Interestingly, but consistent with the concept that proliferation of subcutaneous fat may avert the storage of metabolically harmful “ectopic fat” in the liver and omentum, insulin sensitivity and glycemic control was shown to be superior in individuals seropositive for Ad36 compared with seronegative individuals (13).

Possible mechanisms for infection-induced obesity may involve a peripheral effect on fat cell differentiation and storage or a central effect on appetite (6). Adipose tissue expansion may occur during acute infection only or possibly sustained during latent or chronic illness (14).

An understudied pathogen of potential interest in obesity research is the protozoan parasite *Toxoplasma gondii (T. gondii*). *T. gondii* is a parasite common in both developed and developing countries (15), infecting approximately 30% of the population worldwide (16). Its life cycle involves distinct developmental stages. It takes the form of a rapidly replicating tachyzoite during acute infection and progresses in an immunocompetent host to a slower, relatively dormant growing bradyzoite within tissue cysts. Sexual reproduction or oocyst state can only be achieved if the parasite is transmitted to a feline host (17). The most common cause of human infection is by ingestion of undercooked meat or incomplete washing of contaminated vegetables, but can also occur via accidental inoculation of tachyzoites, organ transplantation, blood transfusion, or transplacental transmission from infected mothers (18). Acute infection may cause minimal (e.g., lymphadenopathy) or no symptoms, and latent infection in an immunocompetent individual is likely to become symptomatic only under conditions of immunosuppression (15). Individuals are not routinely screened for infection unless they are pregnant or immunocompromised.

Experimentally, in rats, *T. gondii* infection was associated with significant weight gain after 30 days of inoculation followed by weight loss over the next 60 days (19). The authors hypothesized that weight gain may have been due to direct central effects, i.e., behavioral changes (e.g., increased food intake) associated with *T. gondii* cysts in the brain and/or indicated central effects, e.g., altered hypothalamic function (e.g., appetite regulation) caused by peripheral tissue inflammation. Weight related effects of *T. gondii* infection may be influenced by strain. In another animal study, two different strains of *T. gondii* had opposite effects on body weight (20).

The only previous study investigating the possible association between *T. gondii* and obesity in humans reported negative results. Thjodleifsson et al. (21) measured *T. gondii* IgG antibodies among individuals participating in the European Community Health Survey I. There was no significant difference in presence of antibodies among individuals who were overweight (BMI ≥25 kg/m^2^) compared to those who were not overweight. A possible factors which may have influenced the lack of association was the exclusion of adults >44 years old, since acquired toxoplasmosis increases with age.

In our study, we investigated the possible association between obesity and toxoplasmosis in a sample of adults (including individuals 18–80 years), in a country (Germany) with a high seroprevalence rate of *T. gondii* infection. We hypothesized that positive *T. gondii* serology will be more common among individuals who are obese compared to those who are non-obese, and that antibody titers will be higher for the obese individuals. We also tested for an association between obesity and seropositivity of two other common latent infections that often co-occur with toxoplasmosis (CMV and HSV1) to see if the association is specific to *T. gondii*.

## Materials and Methods

### Participants

Detailed recruitment methodology is described in Serretti (22). Adult male and female individuals ages 18–80 years old were randomly selected from the Munich, city registry in Germany and invited by mail to participate. Study recruitment began in 1998. Those who responded were screened by phone to obtain medical and psychiatric histories. Individuals whose phone screening was negative were invited to complete a clinic visit where they underwent the Structured Clinical Interview for DSM-IV [SCID I and SCID II; (23–25)] to validate the absence of any lifetime history of mental illness and personality disorders. The participants also underwent a neurological examination to exclude the presence of any current CNS impairment and the Family History Assessment Module (26) was administered to rule out the presence of psychiatric disorders in their first-degree relatives. The Mini Mental State Examination was used to rule out possible cognitive impairment in those older than 60 years. Weight and height was obtained by self-report. Waist circumference was measured at the study visit. Education level, graded according to a three-level variable (1 for secondary school; 2 for junior high school, and 3 for general qualification for university entrance) was used to define socioeconomic status.

The study was conducted in accordance with the ethical standards of the 1964 Declaration of Helsinki and with approval of the local ethics committee in Germany. Written informed consent was obtained from all subjects.

### *T. gondii* serological analysis

Solid phase enzyme immunoassay method was used to test for IgG antibodies to *T. gondii* as previously described (27). This procedure involved exposure of diluted serum to antigens embedded on the wells of microtiter plates. Antibody binding was quantified by reaction labeled anti-human IgG with the enzyme substrate. Quantitative measurement of antibodies (i.e., serointensity) was based on the ratio of the optical density of the blood sample and a standard containing 10 international units of anti-*T. gondii* antibody. Seropositivity was defined as a *T. gondii* IgG titer ≥0.8 (27). The lab analysis was done at Dr. Robert Yolken’s lab at Johns Hopkins University supported by Stanley Medical Research Institute. The technician was blind to the study hypothesis.

#### Data analysis plan

Descriptive statistics included proportions for categorical variables and means, standard deviations for continuous variables; continuous distributions were inspected for outliers and skewing. Proportions were compared by chi-squared tests; means were compared by *t*-tests or one-way ANOVA with LSD *post hoc* comparisons. Logistic regression models were built to assess relative odds for obesity comparing *T. gondii* seropositives with seronegatives. Linear regression models were built to estimate adjusted mean *T. gondii* serum IgG titers by waist circumference. Age, sex, and education were included in models if they changed coefficients by approximately 10% or more, or were significantly associated with the outcome.

## Results

The continuous BMI variable was used to generate three strata of obesity categories – obese (BMI >30 kg/m^2^), overweight (BMI ≤30 or ≥25), and non-obese/overweight (BMI <25 kg/m^2)^. The weight and height given by one participant indicated a BMI of >65, suggesting a rare, genetic cause of obesity. This individual was excluded from all further analyses and tables. Demographics of the participant sample and BMI distribution by *T. gondii* seropositivity status (positive or negative) are presented in Table 1. As expected, there was a significantly increasing trend of seropositivity with age (i.e., increased time for possible environmental exposure). Gender was not associated with obesity. Male and female gender were almost evenly distributed in the entire sample and in both the *T. gondii* positive and negative groups so gender was not adjusted in the analysis. Individuals with higher levels of education had lower odds of obesity compared with those with lower education background. About 7.4% of the sample was obese (BMI ≥30 kg/m^2^).

**Table 1:** **Demographics of study sample**.

	*T. gondii* seropositivity			*p* Value
	Positive *N* = 498	Negative *N* = 501	Combined *N* = 999
	*N* (%)	*N* (%)	*N* (%)	
Sex
Male	255 (51.2)	235 (46.9)	490 (49.1)	0.17
Female	243 (48.8)	266 (53.1)	509 (51.0)	
Education level
1	153 (30.7)	92 (18.4)	245 (24.6)	<0.0001
2	155 (31.1)	147 (29.4)	302 (30.3)	
3	190 (38.2)	261 (52.2)	451 (45.2)	
Age (quartiles)
<40 years	59 (11.9)	179 (35.7)	238 (23.8)	<0.0001
40 to <60	122 (24.5)	141 (28.1)	263 (26.3)	
60 to <67	130 (26.1)	97 (19.4)	227 (22.7)	
≥67	187 (37.6)	84 (16.8)	271 (27.1)	
BMI
Normal <25	265 (53.3)	310 (62.1)	575 (57.7)	0.0001
Overweight <30	179 (36.0)	168 (33.7)	347 (34.8)	
Obese 30+	53 (10.7)	21 (4.2)	74 (7.4)	

*One participant with BMI >65 was omitted as an outlier*.

Individuals who were positive for *T. gondii* IgG had approximately twice the odds of being obese compared to seronegative individuals, adjusting for age (*p* = 0.01) (see Table 2). Further, individuals who were obese had significantly higher levels of IgG than individuals who were overweight or normal (*p* = 0.01). There was no significant difference in IgG titers for individuals who were overweight compared to normal weight. Gender and education (as a surrogate for socioeconomic status) were also assessed for confounding; when entered into the models there was no change in the effects of BMI. Neither CMV nor HSV1 seropositivity was significantly associated with obesity after adjusting for age and education.

**Table 2:** **Age-adjusted odds ratios for obesity (BMI ≥30) compared to overweight and normal participants combined, from logistic regression analysis**.

	Obese vs. non-obese		
	OR	95% CI	*p* Value
*T. gondii* positive vs. negative	1.97	1.15, 3.39	0.01
Age (quartiles)
<40 years	1.00	–	ref
40 to <60 vs. <40	2.40	0.86, 6.70	0.10
60 to <67 vs. <40	3.43	1.24, 9.52	0.02
≥67 vs. <40	3.35	1.22, 9.20	0.02
Sex male vs. female	0.81	0.49, 1.33	0.40
Education
1	1.00	–	ref
2 vs. 1	0.89	0.51, 1.57	0.70
3 vs. 1	0.39	0.20, 0.75	0.005

*1 = secondary education; 2 = junior high school education; 3 = university equivalent*.

## Discussion

To our knowledge, this is the first study to identify an association between *T. gondii* and obesity among individuals carefully screened for mental illness, including personality disorders. Mental illness is an important confounding variable, since obesity rates are higher among individuals with serious mental illness, and latent *T. gondii* infection has been previously associated with schizophrenia (28), bipolar disorder (29), and personality disorder (29). Screening for mental illness, as well as self-selection bias, may have resulted in a relatively lean sample, as only less than 10% of individuals in the study were obese. In contrast, a German study with a large adult sample from a primary care setting that did not screen for mental illness, approximately 24% of males and 23% of women were obese (30).

Because this is an association study, we are unable to determine if there is a causal relationship between *T. gondii* seropositivity and obesity. However, evidence from animal studies indicates possible connections between parasite survival and obesity risk. Parasites may alter host appetite regulation to increase nutrient intake. For example, infection with *Taenia taeniaeformis* is associated with lower levels of leptin (31). Leptin is a peptide appetite hormone that signals satiety so lower levels would stimulate food intake. Parasites may also indirectly increase appetite through behavioral changes. *T. gondii* brain cysts have greater distribution in the amygdala and nucleus accumbens, brain regions that may be primarily targeted to influence fear signaling to promote survival (i.e., reduced vigilance may make infected rodents more susceptible to predator cats who are the definitive host for replication) and these regions also influences reward driven behavior (e.g., eating related behaviors) (32). *T. gondii* may also influence motivation and reward driven behaviors through altered dopamine pathways. Animal models indicate that *T. gondii* influences both dopamine release and availability of the rate limiting enzyme in dopamine synthesis, and dopamine antagonists (i.e., antipsychotic medications) can block behavior changes in *T. gondii* infected rats (32). Thus, parasite induced host behavioral changes that may be driven by survival needs may have inadvertent effects on eating patterns that promote obesity. Parasites also may interfere with host lipid metabolism. *T. gondii* “co-opts” some aspects of host lipid metabolism, specifically tachyzoite acquisition of cholesterol occurs through host cell endocytosis and the low density lipoprotein pathway (33). Thus, parasitic infection may have metabolic effects beyond weight gain.

*Toxoplasma gondii* may also influence obesity risk through alterations in inflammatory pathways. T cells play a major role in *T. gondii* resistance, with mouse models demonstrating a strong Th1 immune response to maintain the dormant bradyzoite stage (34). Th1 immune response is maintained to promote long-lasting immunity (35). Obesity has been previously referred to as “a state of chronic inflammation” (36). In animal studies, mice with diet induced obesity have more T cells in adipose tissue compared to lean control mice (37), and diet controlled weight loss is associated with reduced adipose tissue inflammation (38). Chronic inflammation has been implicated in development of insulin resistance and other metabolic abnormalities (39) and is associated with reduced metabolic rate (40). Thus, the chronic, pro-inflammatory state induced by latent *T. gondii* infection may either precipitate (in non-obese individuals), perpetuate, or exacerbate (in obese people) the inflammation related weight gain. In contrast, certain intestinal parasitic infections with nematodes induce a Th2-polarized immune response and are associated with weight loss (41), suggesting the possible association between body habitus and pathogen is specific to the type of immune response and the specific infectious agent. Of note, we did not find an association between obesity and HSV or CMV1 seropositivity.

The association between *T. gondii* and obesity, if replicated, has important public health significance because of the high prevalence of *T. gondii* worldwide. The CDC has prioritized *T. gondii* as one of the top “Five Neglected Parasitic Infections” based on its high prevalence in the US, severity of illness, and potential for prevention (http://www.cdc.gov/parasites/npi.html; accessed 9-25-2013). Food-borne infections have continued to increase in recent years, with increased consumer preference for exotic and global food sources and public demand for free-range livestock, which drives greater exposure to environmental soil spread pathogens (42). Possible public health interventions to reduce *T. gondii* infection include vaccine development (43), interventions (44), as well as efforts to reduce unsafe food handling processes.

### Limitations

The cross sectional nature of the study does not allow us to investigate possible causal relationships between *T. gondii* and obesity, including a possible reverse causality (i.e., obesity promoting the risk of *T. gondii* infection) or shared causality (i.e., a common factor causing both obesity and *T. gondii* infection). However, this association, if replicated, supports the need for further research to investigate the direction (i.e., does obesity increase risk of latent infection or vice versa) and mechanisms that that drive this relationship. Interventions that target host lipid metabolism, for example, have been suggested as a novel intervention target for *T. gondii* infection since this pathway is important for parasite survival. (33) Such interventions may have the advantage of both reducing risk of infection and promoting metabolic health.

Another limitation of the study is that weight (to determine BMI) was provided by self-report. Interestingly, the Survey of Health, Aging, and Retirement in Europe 2004 (SHARE) study that interviewed individuals >50 years old from over 10 European countries indicated that Germans tended to “under-rate” their health on self-report forms compared to respondents from other countries (45), and in another European study with a large primary care sample indicates that estimate BMI varied from true measure by only +0.19 for men and +0.17 for women, suggesting that self-report may be adequate for monitoring national obesity prevalence (46).

## Conclusion

This study identified a positive relationship between *T. gondii* seropositivity and obesity in a relatively large sample carefully screened for mental illness. Future studies are needed to examine the relationship between obesity and *T. gondii* titers over time to investigate possible causality and persistence of this association over time. Research is also needed to further assess possible association between *T. gondii* and other metabolic parameters. Serotyping could help determine if metabolic effects in humans differ with different strains of pathogen. *T. gondii* latent infection may turn out to have excellent potential as a target for public health interventions because of the high prevalence of this condition, in both developed and underdeveloped countries, and the possibility of identifying novel targets for obesity interventions.

## Conflict of Interest Statement

The authors declare that the research was conducted in the absence of any commercial or financial relationships that could be construed as a potential conflict of interest.

## References

[1] Kelley T Yang W Chen CS Reynolds K He J Global burden of obesity in 2005 and projections to 2030. Int J Obes (2008) 32(9):1431–7 10.1038/ijo.2008.102 18607383

[2] Wang C Coups EJ Causal beliefs about obesity and associated health behaviors: results from a population-based survey. Int J Behav Nutr Phys Act (2010) 7:19 10.1186/1479-5868-7-19 20199677 PMC2842229

[3] Rao G Burke LE Spring BJ Ewing LJ Turk M Lichtenstein AH New and emerging weight management strategies for busy ambulatory settings: a scientific statement from the American Heart Association endorsed by the Society of Behavioral Medicine. Circulation (2011) 124(10):1182–203 10.1161/CIR.0b013e31822b9543 21824925

[4] Mann T Tomiyama AJ Westling E Lew AM Samuels B Chatman J Medicare’s search for effective obesity treatments: diets are not the answer. Am Psychol (2007) 62(3):220–33 10.1037/0003-066X.62.3.220 17469900

[5] Dhurandhar NV Infectobesity: obesity of infectious origin. J Nutr (2001) 131(10):2794S–7S 11584109 10.1093/jn/131.10.2794S

[6] Vasilakopoulou A le Roux CW Could a virus contribute to weight gain? Int J Obes (Lond) (2007) 31(9):1350–6 10.1038/sj.ijo.0803623 17420782

[7] Zupancic ML Cantarel BL Liu Z Drabek EF Ryan KA Cirimotich S Analysis of the gut microbiota in the old order Amish and its relation to the metabolic syndrome. PLoS One (2012) 7(8):e43052 10.1371/journal.pone.0043052 22905200 PMC3419686

[8] Dhurandhar NV A framework for identification of infections that contribute to human obesity. Lancet Infect Dis (2011) 11(12):963–9 10.1016/S1473-3099(11)70274-2 22115071

[9] Lyons MJ Faust IM Hemmes RB Buskirk DR Hirsch J Zabriskie JB A virally induced obesity syndrome in mice. Science (1982) 216(4541):82–5 10.1126/science.7038878 7038878

[10] Esposito S Preti V Consolo S Nazzari E Principi N Adenovirus 36 infection and obesity. J Clin Virol (2012) 55(2):95–100 10.1016/j.jcv.2012.06.009 22771001

[11] van Ginneken V Sitnyakowsky L Jeffery JE Infectobesity: viral infections (especially with human adenovirus-36: Ad-36) may be a cause of obesity. Med Hypotheses (2009) 72(4):383–8 10.1016/j.mehy.2008.11.034 19138827

[12] Yamada T Hara K Kadowaki T Association of adenovirus 36 infection with obesity and metabolic markers in humans: a meta-analysis of observational studies. PLoS One (2012) 7(7):e42031 10.1371/journal.pone.0042031 22848697 PMC3405004

[13] Lin WY Dubuisson O Rubicz R Liu N Allison DB Curran JE Long-term changes in adiposity and glycemic control are associated with past adenovirus infection. Diabetes Care (2013) 36(3):701–7 10.2337/dc12-1089 23160725 PMC3579356

[14] McAllister EJ Dhurandhar NV Keith SW Aronne LJ Barger J Baskin M Ten putative contributors to the obesity epidemic. Crit Rev Food Sci Nutr (2009) 49(10):868–913 10.1080/10408390903372599 19960394 PMC2932668

[15] Flegr J Influence of latent toxoplasma infection on human personality, physiology, and morphology: pros and cons of the toxoplasma-human model in studying the manipulation hypothesis. J Exp Biol (2013) 216(Pt 1):127–33 10.1242/jeb073635 23225875

[16] Flegr J Stríž I Potential immunomodulatory effects of latent toxoplasmosis in humans. BMC Infect Dis (2011) 11:274 10.1186/1471-2334-11-274 22008411 PMC3213179

[17] Skariah S McIntyre MK Mordue DG *Toxoplasma gondii*: determinants of tachyzoite to bradyzoite conversion. Parasitol Res (2010) 107(2):253–60 10.1007/s00436-010-1899-6 20514494 PMC3327608

[18] Tenter AM Heckeroth AR Weiss LM *Toxoplasma gondii*: from animals to humans. Int J Parasitol (2000) 30(12–13):1217–58 11113252 10.1016/s0020-7519(00)00124-7 PMC3109627

[19] Hermes-Uliana C Pereira-Severi LS Luerdes RB Franco CL da Silva AV Araújo EJ Chronic infection with *Toxoplasma gondii* causes myenteric neuroplasticity of the jejunum in rats. Auton Neurosci (2011) 160(1–2):3–8 10.1016/j.autneu.2010.09.003 20932812

[20] Kannan G Moldovan K Xiao JC Yolken RH Jones-Brando L Pletnikov MV *Toxoplasma gondii* strain-dependent effects on mouse behaviour. Folia Parasitol (Praha) (2010) 57(2):151–5 20608478 10.14411/fp.2010.019

[21] Thjodleifsson B Olafsson I Gislason D Gislason T Jögi R Janson C Infections and obesity: a multinational epidemiological study. Scand J Infect Dis (2008) 40(5):381–6 10.1080/00365540701708293 17943636

[22] Serretti A Mandelli L Giegling I Schneider B Hartmann AM Schnabel A HTR2C and HTR1A gene variants in German and Italian suicide attempters and completers. Am J Med Genet B Neuropsychiatr Genet (2007) 144B(3):291–9 17192951 10.1002/ajmg.b.30432

[23] First MB Spitzer RL Gibbon M Williams BW Benjamin L Structured Clinical Interview for DSM-IV Axis II Personality Disorders (SCID-II). New York: New York State Psychiatric Institute (1990).

[24] First MD Spitzer RL Gibbon M Williams JB Structured Clinical Interview for DSM-IV Axis I Disorders – Patient Edition (SCID-I/p, Version 2.0). New York: New York State Psychiatric Institute (1995).

[25] Wittchen H Zaudig M Fydrich T Strukturiertes Klinisches Interview fur DSM-IV. Gottingen: Achse I und llHogrefe (1997).

[26] Rice JP Reich T Bucholz KK Neuman RJ Fishman R Rochberg N Comparison of direct interview and family history diagnoses of alcohol dependence. Alcohol Clin Exp Res (1995) 19(4):1018–23 10.1111/j.1530-0277.1995.tb00983.x 7485811

[27] Arling TA Yolken RH Lapidus M Langenberg P Dickerson FB Zimmerman SA *Toxoplasma gondii* antibody titers and history of suicide attempts in patients with recurrent mood disorders. J Nerv Ment Dis (2009) 197(12):905–8 10.1097/NMD.0b013e3181c29a23 20010026

[28] Torrey EF Bartko JJ Lun ZR Yolken RH Antibodies to *Toxoplasma gondii* in patients with schizophrenia: a meta-analysis. Schizophr Bull (2007) 33(3):729–36 10.1093/schbul/sbl050 17085743 PMC2526143

[29] Hinze-Selch D Däubener W Erdag S Wilms S The diagnosis of a personality disorder increases the likelihood for seropositivity to *Toxoplasma gondii* in psychiatric patients. Folia Parasitol (Praha) (2010) 57(2):129–35 20608475 10.14411/fp.2010.016

[30] Hauner H Bramlage P Losch C Steinhagen-Thiessen E Schunkert H Wasem J Prevalence of obesity in primary care using different anthropometric measures – results of the German metabolic and cardiovascular risk projects (GEMCAS). BMC Public Health (2008) 8:282 10.1186/1471-2458-8-282 18694486 PMC2518927

[31] Lõhmus M Moalem S Björklund M Leptin, a tool of parasites? Biol Lett (2012) 8(5):849–52 10.1098/rsbl.2012.0385 22740641 PMC3440990

[32] Prandovszky E Gaskell E Martin H Dubey JP Webster JP McConkey GA The neurotropic parasite *Toxoplasma gondii* increases dopamine metabolism. PLoS One (2011) 6(9):e23866 10.1371/journal.pone.0023866 21957440 PMC3177840

[33] Boyle JP Radke JR A history of studies that examine the interactions of toxoplasma with its host cell: emphasis on in vitro models. Int J Parasitol (2009) 39(8):903–14 10.1016/j.ijpara.2009.01.008 19630139

[34] Denkers EY Gazzinelli RT Regulation and function of T-cell-mediated immunity during *Toxoplasma gondii* infection. Clin Microbiol Rev (1998) 11(4):569–88 9767056 10.1128/cmr.11.4.569 PMC88897

[35] Gigley JP Fox BA Bzik DJ Cell-mediated immunity to *Toxoplasma gondii* develops primarily by local Th1 host immune responses in the absence of parasite replication. J Immunol (2009) 182(2):1069–78 19124750 10.4049/jimmunol.182.2.1069 PMC2615398

[36] Dandona P Aljada A Bandyopadhyay A Inflammation: the link between insulin resistance, obesity and diabetes. Trends Immunol (2004) 25(1):4–7 10.1016/j.it.2003.10.013 14698276

[37] Rocha VZ Folco EJ Sukhova G Shimizu K Gotsman I Vernon AH Interferon-gamma, a Th1 cytokine, regulates fat inflammation: a role for adaptive immunity in obesity. Circ Res (2008) 103(5):467–76 10.1161/CIRCRESAHA.108.177105 18658050 PMC2740384

[38] Wang Q Perrard XD Perrard JL Mansoori A Raya JL Hoogeveen R Differential effect of weight loss with low-fat diet or high-fat diet restriction on inflammation in the liver and adipose tissue of mice with diet-induced obesity. Atherosclerosis (2011) 219(1):100–8 10.1016/j.atherosclerosis.2011.07.025 21824616 PMC3206136

[39] Priceman SJ Kujawski M Shen S Cherryholmes GA Lee H Zhang C Regulation of adipose tissue T cell subsets by Stat3 is crucial for diet-induced obesity and insulin resistance. Proc Natl Acad Sci U S A (2013) 110(32):13079–84 10.1073/pnas.1311557110 23878227 PMC3740863

[40] Gregor MF Hotamisligil GS Inflammatory mechanisms in obesity. Annu Rev Immunol (2011) 29:415–45 10.1146/annurev-immunol-031210-101322 21219177

[41] Wong T Hildebrandt MA Thrasher SM Appleton JA Ahima RS Wu GD Divergent metabolic adaptations to intestinal parasitic nematode infection in mice susceptible or resistant to obesity. Gastroenterology (2007) 133(6):1979–88 10.1053/j.gastro.2007.09.006 18054569 PMC2180166

[42] Newell DG Koopmans M Verhoef L Duizer E Aidara-Kane A Sprong H Food-borne diseases – the challenges of 20 years ago still persist while new ones continue to emerge. Int J Food Microbiol (2010) 139(Suppl 1):S3–15 10.1016/j.ijfoodmicro.2010.01.021 20153070 PMC7132498

[43] Mendes ÉA Fonseca FG Casério BM Colina JP Gazzinelli RT Caetano BC Recombinant vaccines against *T. gondii*: comparison between homologous and heterologous vaccination protocols using two viral vectors expressing SAG1. PLoS One (2013) 8(5):e63201 10.1371/journal.pone.0063201 23690999 PMC3654925

[44] Hartmann K Addie D Belák S Boucraut-Baralon C Egberink H Frymus T *Toxoplasma gondii* infection in cats: ABCD guidelines on prevention and management. J Feline Med Surg (2013) 15(7):631–7 10.1177/1098612X13489228 23813830 PMC11148961

[45] Jürges H True health vs response styles: exploring cross-country differences in self-reported health. Health Econ (2007) 16(2):163–78 10.1002/hec.1134 16941555

[46] Bolton-Smith C Woodward M Tunstall-Pedoe H Morrison C Accuracy of the estimated prevalence of obesity from self reported height and weight in an adult Scottish population. J Epidemiol Community Health (2000) 54(2):143–8 10.1136/jech.54.2.143 10715748 PMC1731630

